# Trends in the burden of urolithiasis in China: an analysis from the global burden of disease study 2021

**DOI:** 10.3389/fsurg.2025.1537706

**Published:** 2025-02-18

**Authors:** Yangyang Lin, Qing-cheng Lin, Qing-ying Zhou, Nai-fen Xu, Ding-qin Zheng, Xin-jun Wang, Ran Xu

**Affiliations:** ^1^ Department of Urology, Pingyang Hospital of Wenzhou Medical University, Wenzhou, China; ^2^ Department of Xiaojiang, Pingyang Hospital of Wenzhou Medical University, Wenzhou, China; ^3^ Department of Urology, Zhongshan Hospital Xiamen University, School of Medicine, Xiamen University, Xiamen, China

**Keywords:** urolithiasis, global burden of disease, Joinpoint regression, age-period-cohort analysis, prevalence

## Abstract

**Background:**

Urolithiasis is a common disease of the urinary tract, the global prevalence of which is increasing year by year and which, due to its high rate of recurrence and complications, represents a major burden on the quality of life of patients and on the global public health system. As the most populous country in the world, the epidemiology of urolithiasis in China is of great importance. However, the current systematic epidemiological assessment of urolithiasis in China is relatively limited. Therefore, this study used the GBD 2021 database to systematically assess the disease burden of urolithiasis in China to provide a basis for policy formulation.

**Methods:**

This study analysed the disease burden of urolithiasis in China between 1992 and 2021, including the number of prevalence cases, prevalence rate and age-standardised prevalence rate, using data from the GBD 2021 database. Joinpoint regression models were used to identify changes in the annual trends of urolithiasis, using annual percent change and average annual percent change for description. Age-period-cohort and Bayesian age-period-cohort models were used to assess time trends in urolithiasis burden and to predict trends over the next 15 years, respectively.

**Result:**

The age-standardised prevalence rate of urolithiasis in China has decreased from 96.23 per 100,000 in 1992 to 50.78 per 100,000 in 2021 for males and from 34.44 per 100,000 in 1992 to 22.04 per 100,000 in 2021 for females. While the number of men with the disease has declined slightly, the number of women with the disease has increased. The Joinpoint regression model showed that the age-standardised prevalence rate showed a consistent downward trend in both males and females, and that the periods in which the decline was most pronounced were very similar. The age-period-cohort model also confirmed that the period and cohort effects of urolithiasis showed a decreasing trend from year to year. In addition, the age effect suggested that the risk of urolithiasis tended to increase and then decrease with age, and that the risk was highest in the 55–59 age group. Finally, the Bayesian age-period-cohort prediction model showed that the age-standardised prevalence rate of urolithiasis in both males and females would show a slowly increasing trend over the next 15 years.

**Conclusion:**

In this study, we analysed the trend of the disease burden of urolithiasis in China during 1992–2021 by GBD 2021. The results showed that the burden of urolithiasis was significantly higher in males than in females. Furthermore, although the burden of urolithiasis has gradually improved in both men and women over the past 30 years, the BAPC prediction model suggests that the burden of urolithiasis is likely to increase in the next 15 years in both sexes. Therefore, prevention, early screening and treatment of urolithiasis in high-risk groups need to be strengthened to respond effectively to a possible future increase in burden.

## 1 Introduction

Urolithiasis, defined as the formation of stones in the upper or lower urinary tract, is one of the most common diseases of the urinary system. Epidemiological studies have shown that the prevalence of urolithiasis varies from continent to continent and country to country, ranging from 1% to 20% (1), due to differences in social conditions, dietary habits, climate and other factors. At the same time, the prevalence of urolithiasis has been found to be increasing worldwide (2).

Urolithiasis is characterised by a variety of symptoms including infection, pain and haematuria. Until now, surgical treatments, including shock wave lithotripsy, ureteroscopy and percutaneous nephrolithotripsy, have been considered the most effective way to treat urolithiasis (1). However, urolithiasis has the disease characteristic of easy recurrence, and the recurrence rate can be as high as 50% according to relevant research statistics (3), so the incidence of repeated surgical interventions in urolithiasis is high. In addition, the symptoms and high recurrence rate of urolithiasis not only greatly affect patients' quality of life, but also increase the risk of renal failure, osteoporosis, gingivitis and other complications (4–6). Thus, given the characteristics of the disease and its increasing prevalence, urolithiasis has become an enormous burden on global public health.

As the world's most populous country and an important link in the promotion of a global community of health for all, China plays a pivotal role in global public health. Therefore, understanding the prevalence of urolithiasis in China is important for the global management of urolithiasis. On the other hand, systematic and scientific epidemiological studies play a crucial role in assessing the burden of disease and formulating related policies.

As a major programme of the Institute for Health Metrics and Evaluation (IHME), the Global Burden of Disease (GBD) study has become an important tool for the governance of global public health systems since its first publication in the World Development Report (7, 8). Therefore, this study was designed to make a systematic assessment and systematic prediction of the disease burden of urolithiasis in China in the epidemiological direction using the GBD 2021 database.

## 2 Methods

### 2.1 Data resources and definitions

The GBD 2021 contains data on 371 diseases and injuries and their associated 88 major risk factors from 204 countries and regions. In this study, we obtained data on the burden of disease associated with urolithiasis in China from this database, including the number of prevalence cases, prevalence rates and age-standardised prevalence rates (ASPR) between 1992 and 2021. These data are available online via the website (http://ghdx.healthdata.org/gbd-results-tool).

The GBD data is derived from a mix of direct and indirect sources, including national health surveys, statistical modeling, and expert input, to estimate disease burden, particularly in areas with insufficient data. The sampling approach used by the GBD database differs depending on the available data in each country or region. In nations with well-established vital registration systems, mortality and morbidity data are reported directly. In areas where such data is lacking, statistical models are employed to estimate the disease burden, incorporating information from household surveys, hospital records, and expert contributions.

In the 11th edition of the International Classification of Diseases (ICD-11), urolithiasis is described as a disease of the urinary system caused by dehydration, decreased urine or fluid flow, or increased excretion of minerals such as calcium, oxalate, magnesium, cystine and phosphate. It is characterised by the presence of stones that originate from or are located in the urinary system. The diagnosis may be confirmed by an abdominal x-ray or an intravenous pyelogram. Codes for urolithiasis include upper urinary tract stones (GB70), lower urinary tract stones (GB71) and urolithiasis not otherwise specified (GB7Z).

### 2.2 Joinpoint regression analysis

Joinpoint regression is a commonly used statistical method for analysing local trends in disease. By constructing a regression model and analysing the points of change in trends in time-series data, the method is able to separate and analyse the overall trend into subtrends (9–11). For this reason, the Joinpoint regression model is often cited in public health and epidemiological studies. Annual percent change (APC) and average annual percent change (AAPC) were used to describe the results of Joinpoint regression analyses, and a *p*-value of less than 0.05 was considered statistically significant.

### 2.3 Age-period-cohort analysis

In epidemiology and demography, age, period and cohort are three different factors commonly associated with time. The age-period-cohort (APC) model is a statistical analysis that examines the effects of these three factors on a given outcome (12). Specifically, the age effect refers to the effect on an outcome due to the increasing age of an individual organism; the period effect refers to the set of simultaneous effects on all age groups in a given period, i.e., it reflects the impact of changing trends or events in society as a whole on the population; and the cohort effect reflects the effect on an outcome due to a change in the mode of birth or different exposures of groups born in different generations (13). An online tool (https://analysistools.cancer.gov/apc/) is available to help us perform APC analyses of urolithiasis (14).

### 2.4 BAPC prediction models

The Bayesian Age-Period-Cohort (BAPC) model is an important predictive tool in epidemiological analyses for assessing and predicting temporal trends in disease burden. It combines the strengths of Bayesian statistical methods to better account for the linear dependence between the three effects when analysing predictions of the effects of age, period and birth cohort on an event in population data (15).

### 2.5 Statistical metrics

All of the above analyses were mainly performed using R software (version 4.2.3). The statistical results of this study were expressed as uncertainty intervals (UI), confidence intervals (CI) and relative risks (RR). The UI, defined on the GBD website as “a range of values reflecting the certainty of an estimate”, was used in this study to compare the burden of disease between 1992 and 2021. The CI was used in the Joinpoint, APC and BAPC models. Finally, the RR can be used to describe the general results of the APC model.

## 3 Results

### 3.1 Description of the disease burden

**Figure 1:**
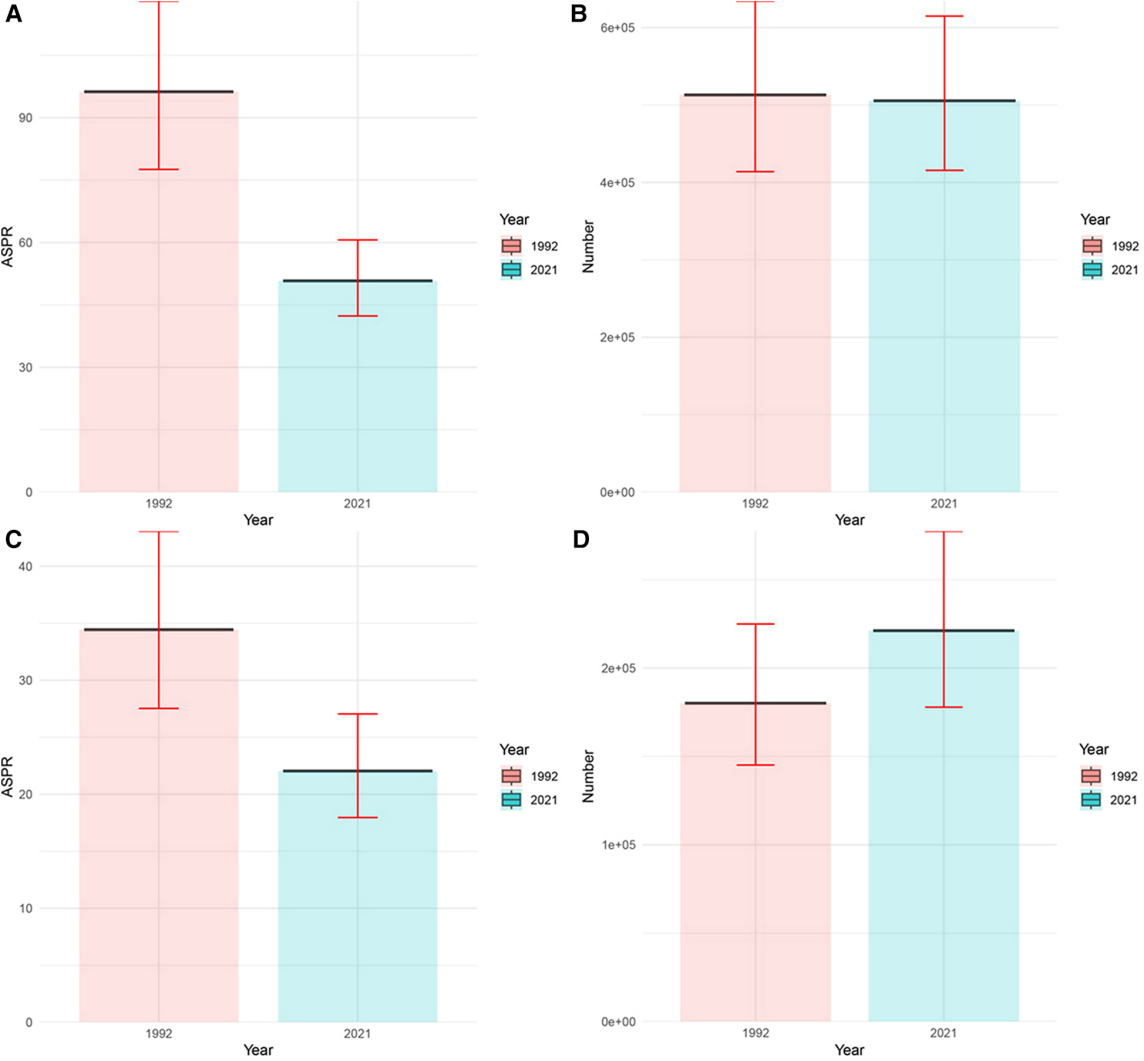
Prevalence of urolithiasis in China in 1992 and 2021. **(A)** ASPR of male; **(B)** number of male; **(C)** ASPR of female; **(D)** number of female.

First, from a male perspective (Figures 1A,B and Supplementary Table S1), the ASPR decreased from 96.23 per 100,000 (95% UI: 77.58–118.01) in 1992 to 50.78 per 100,000 (95% UI: 42.35–60.62) in 2021. In contrast to the marked decrease in the ASPR, the decrease in the number of prevalence cases is more modest, falling only from 512.94 thousand per 100,000 (95% UI: 413.90–634.05 thousand) in 1992 to 505.35 thousand per 100,000 (95% UI: 415.59 to 614.77 thousand) in 2021. On the other hand, for Chinese females (Figures 1C,D and Supplementary Table S1), the ASPR for urolithiasis declined as markedly as for males, from 34.44 per 100,000 (95% UI: 27.53–43.07) in 1992 to 22.04 per 100,000 (95% UI: 17.96–27.05) in 2021. However, the number of prevalence cases among females is quite different from that of males, rising from 180.08 thousand per 100,000 (95% UI: 145.16–224.91 thousand) in 1992 to 221.10 thousand per 100,000 (95% UI: 177.85–277.73 thousand) in 2021.

**Figure 2:**
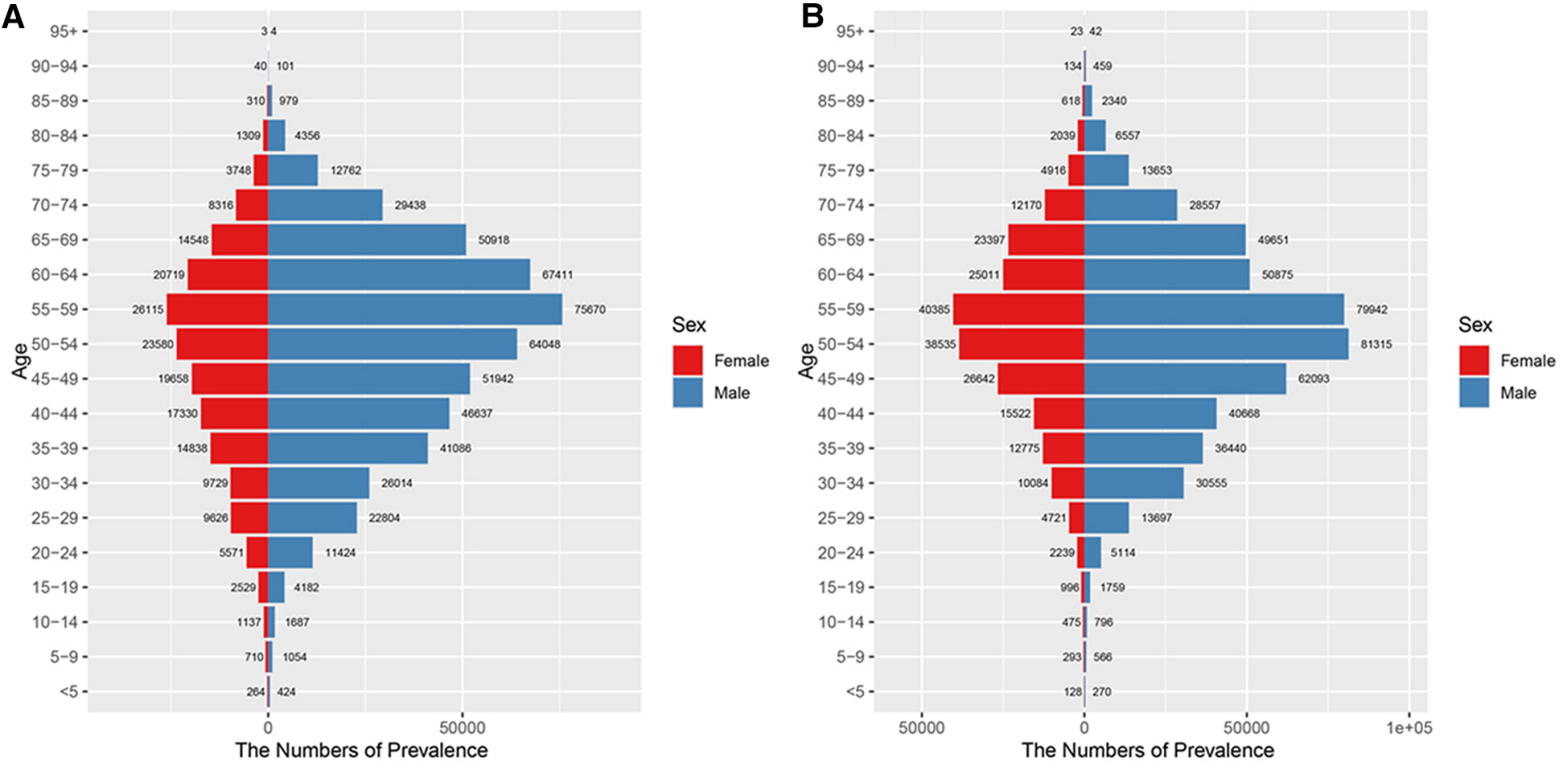
Number of prevalence of urolithiasis in different age groups. **(A)** 1992; **(B)** 2021.

As shown in Figure 2A and Supplementary Table S2, the prevalence of urolithiasis is significantly higher in males than in females, irrespective of age group. Furthermore, the prevalence in both males and females increases and then decreases with age, with the highest number of cases in the 55–59 age group (75.67 thousand for males and 26.12 thousand for females). In addition, as shown in Figure 2B, compared with 1992, the prevalence of urolithiasis in China in 2021 does not show a significant change in the overall trend, i.e., there are still significantly more males than females, and the number of cases shows an increasing and then decreasing trend with age. The difference in the burden of disease between 2021 and 1992 is that the age group with the highest number of cases of urolithiasis in males in 2021 is 50–54 years, whereas in 1992 the age group with the highest number of cases was 55–59 years.

**Figure 3:**
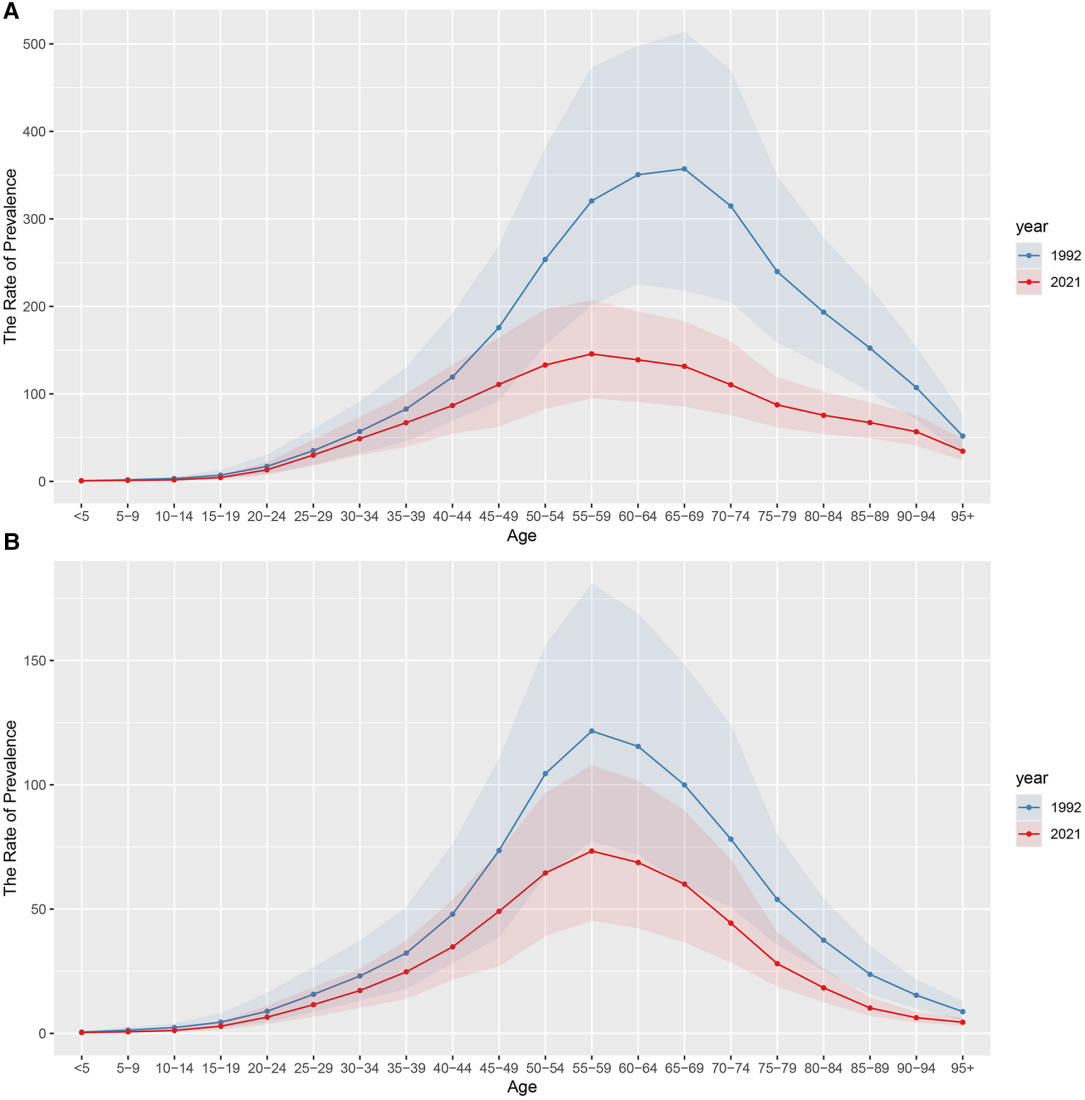
The rate of prevalence of urolithiasis in different age groups. **(A)** male; **(B)** female.

For both males and females, the prevalence rates in 1992 and 2021 show an increasing and then decreasing trend with age (Figure 3 and Supplementary Table S2). In addition, the prevalence rates in 2021 are lower than in 1992 for both males and females. For males, the largest decrease is in the 65–69 age group (225.56 per 100,000), while for females the largest decrease is in the 55–59 age group (48.28 per 100,000). On the other hand, for males, the age group with the highest prevalence rates was 65–69 years in 1992 and 55–59 years in 2021. For females, the age group with the highest prevalence rates is 55–59 years in both 1992 and 2021.

### 3.2 Analysis of the Joinpoint regression model

**Figure 4:**
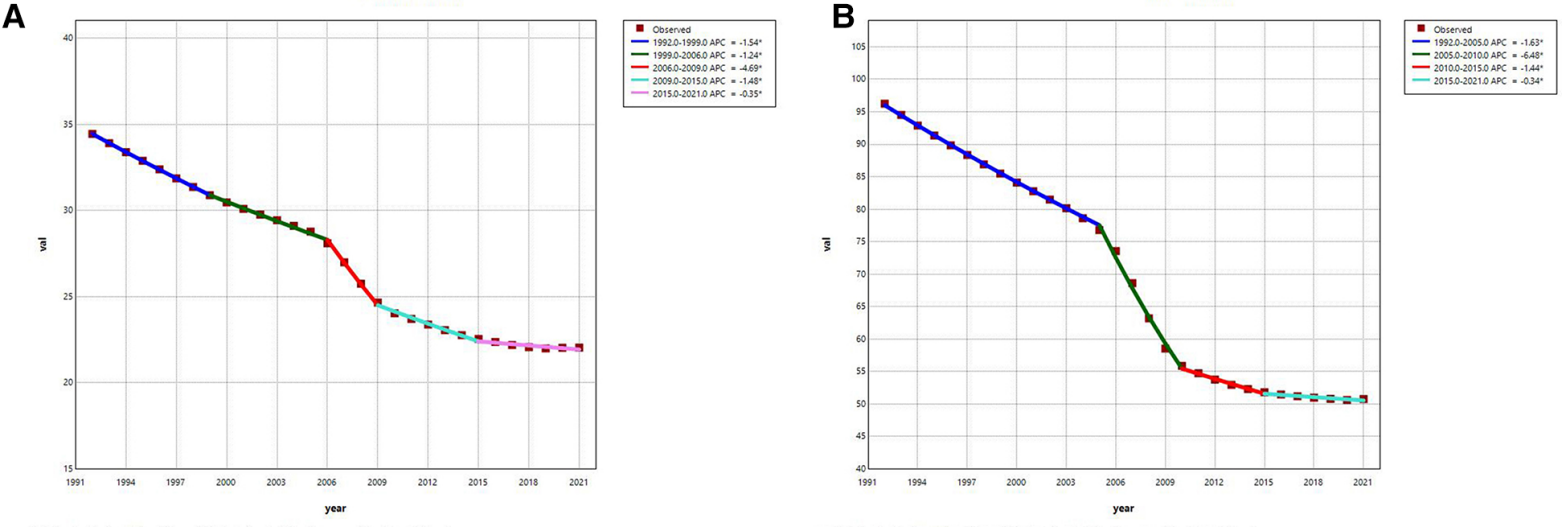
Joinpoint regression analysis: trends of ASPR for urolithiasis in China from 1992 to 2021. **(A)** female; **(B)** male.

Using the Joinpoint regression model, we analysed and visualised the trends in the ASPR of urolithiasis in China between 1992 and 2021 (Figure 4 and Table 1). Firstly, the ASPR of urolithiasis in both females (Figure 4A) and males (Figure 4B) gradually decreased over time, and the *p*-values of the AAPC for both were statistically significant (*p* < 0.001). Second, the overall trend in females was decomposed into five localised trends, and the *p*-values for the APCs of the localised trends were all statistically significant (*p* < 0.001). Among these localised trends, the most significant downward trend was observed between 2006 and 2009, while the opposite was true between 2015 and 2021, where the downward trend was least significant. On the other hand, the overall trend for males was decomposed into four localised trends and all their *p*-values for APC were statistically significant. Furthermore, similar to females, the trend in ASPR for males was most significant between 2005 and 2010 and least significant between 2015 and 2021.

**Table 1:** Joinpoint regression analysis: APC and AAPC of ASPR for urolithiasis in China.

	Year	APC/AAPC (%)	95% CI	Test statistic (*t*)	*p*-Value
Female	1992–1999	−1.54	−1.63 to −1.46	−37.38	<0.001
	1999–2006	−1.24	−1.36 to −1.13	−22.76	<0.001
	2006–2009	−4.69	−5.31 to −4.07	−15.74	<0.001
	2009–2015	−1.48	−1.61 to −1.35	−23.54	<0.001
	2015–2021	−0.35	−0.46 to −0.25	−7.41	<0.001
	1992–2021	−1.54	−1.62 to −1.47	−39.61	<0.001
Male	1992–2005	−1.63	−1.66 to −1.60	−97.80	<0.001
	2005–2010	−6.48	−6.67 to −6.29	−70.34	<0.001
	2010–2015	−1.44	−1.61 to −1.26	−16.95	<0.001
	2015–2021	−0.34	−0.43 to −0.24	−7.47	<0.001
	1992–2021	−2.19	−2.24 to −2.14	−88.09	<0.001

AAPC, average annual percent change presented for full period; APC, annual percent change; CI, confidence interval.

### 3.3 Analysis of APC model

**Figure 5:**
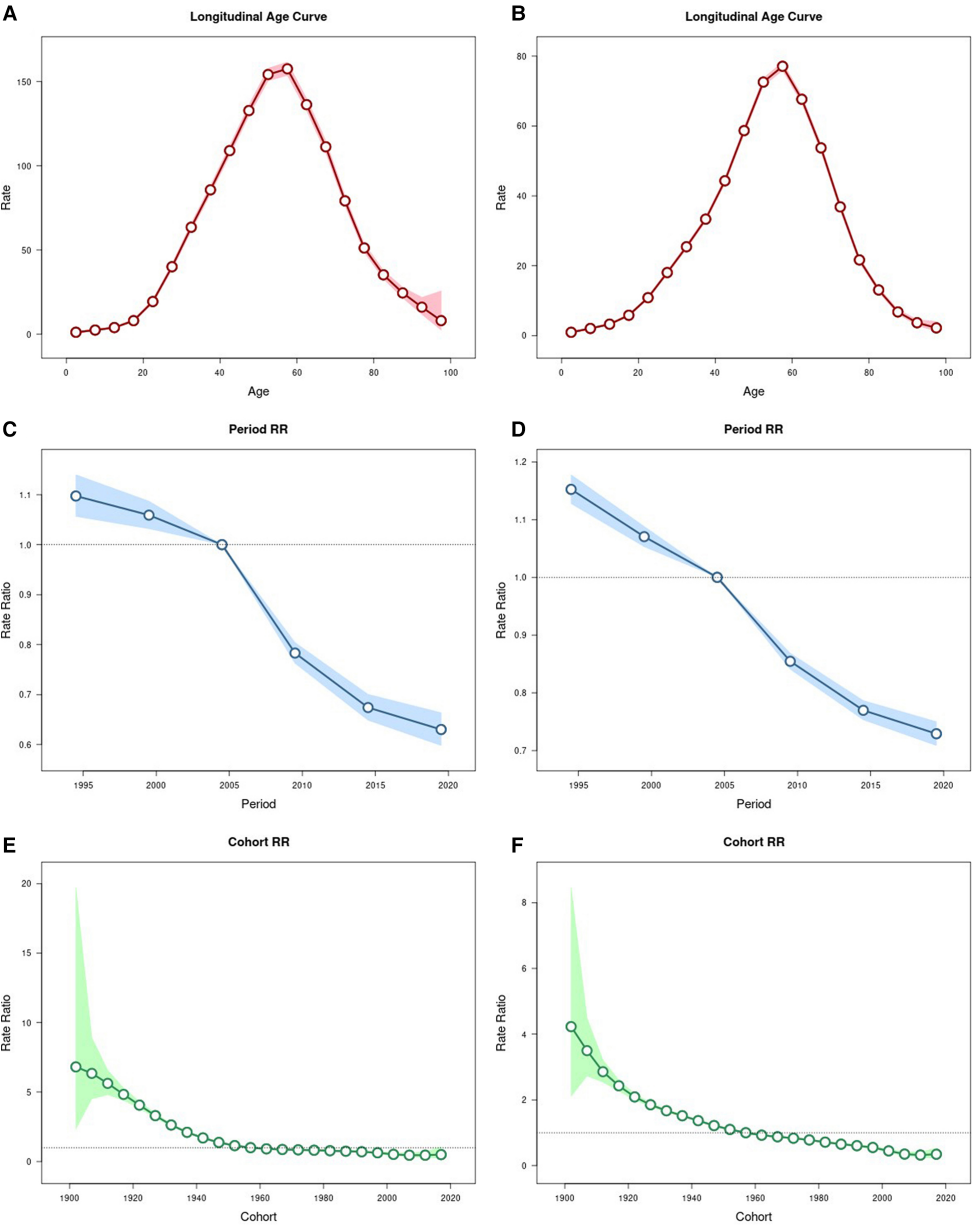
Parameter estimates for the effect of age, period and cohort effects on prevalence rate of urolithiasis. **(A)** age effect on male; **(B)** age effect on female; **(C)** period effect on male; **(D)** period effect on female; **(E)** cohort effect on male; **(F)** cohort effect on female.

In this study, we analysed the effect of three factors, namely age, cycle and cohort, on urolithiasis using the APC model. (Data are shown in Supplementary Tables S3–S5). Firstly, Figures 5A,B illustrate the age effect on urolithiasis in males and females respectively. According to Figures 5A,B, we can see that the prevalence rate in both males and females has a clear peak, i.e., it shows a trend of increasing and then decreasing with age. Not only that, the peak risk was observed in the age group 55–59 years for males (RR = 157.62; 95% CI = 153.73–161.61), but also for females (RR = 77.10; 95% CI = 75.91–78.30). On the other hand, Figures 5C,D suggest the influence of the period effect on urolithiasis in males and females respectively. Similar to the age effect, the trend of the period effect on urolithiasis was relatively similar in males and females, with both sexes showing a decreasing trend in the risk of the disease over time. For men, the RR = 1.10 (95% CI = 1.06–1.14) and RR = 0.63 (95% CI = 0.60–0.66) for the periods 1992–1996 and 2017–2021, respectively. For women, RR = 1.15 (95% CI = 1.12–1.18) and RR = 0.73 (95% CI = 0.71–0.75) for the periods 1992–1996 and 2017–2021, respectively. As for the cohort analyses, Figures 5E,F suggest that the closer the cohort is to 2021, the lower the risk of urolithiasis prevalence for both men and women.

### 3.4 Predictive analyses of the BAPC model

**Figure 6:**
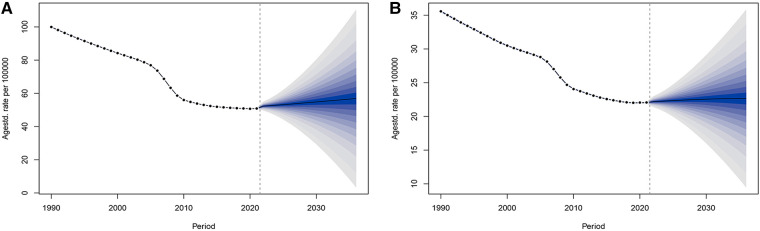
Predicted trends in ASPR for urolithiasis in China over the next 15 years. **(A)** male; **(B)** female.

The ASPR for urolithiasis in males followed similar epidemiological trends to the ASPR for urolithiasis in females as analysed by the BAPC prediction model (Figure 6 and Supplementary Table S6). The ASPR for urolithiasis in both males and females will show a continuous but slow increasing trend over the next 15 years, from 52.29 per 100,000 (95% CI: 49.76–54.82) in 2022 to 56.87 per 100,000 (95% CI: 3.61–110.13) in 2036 for males. For females, it will increase from 22.18 per 100,000 (95% CI: 21.44–22.93) in 2022 to 22.65 per 100,000 (95% CI: 9.54–35.76) in 2036.

## 4 Discussion

By using statistical methods such as Joinpoint regression model, APC model and BAPC prediction model, this study analyses and predicts the disease burden of urolithiasis in China derived from GBD2021, which will help to improve the diagnosis and treatment strategies of urolithiasis in China, thus helping policy makers to make effective decisions.

Comparing the data on the burden of disease in 2021 with that of 1992, the following phenomena can be observed. First, both the ASPR and the number of cases are significantly higher for men than for women. Secondly, the ASPR for men decreases significantly, while the number of prevalence cases for men decreases only slightly. On the other hand, the ASPR for women decreased significantly, while the number of prevalence cases for women increased. These phenomena can be attributed to the following reasons:

(1) Differences in biological basis and lifestyle may be the main factors contributing to the higher prevalence burden of urolithiasis in men. Firstly, testosterone has also been shown to affect the risk of stone formation through a number of pathways (16–18). In addition, many in the male population suffer from inadequate water intake due to lifestyle, work pressures and drinking habits, particularly men who work hard in manual jobs and those with heavy drinking habits (19, 20), which can increase urine concentration and thus the risk of urolithiasis.
(2) The inconsistency between the decrease in ASPR and the change in prevalence in men may be related to factors such as the progress of severe aging, the improvement of early diagnosis, and the improvement of lifestyle in China. As China's population ages (21), more older men are entering the high-risk group. Despite the apparent decline in ASPR in men, as the population base of older men gradually increases, the prevalence in this high-risk group will inevitably drag down the trend of improvement in the number of prevalence cases for men as a whole. On the other hand, with increased health awareness and some lifestyle changes (e.g., diet, exercise, etc.) in the male population (22, 23), the prevalence of urolithiasis has decreased in some groups of men. However, the large total number of men in the male population and the chronic and recurrent nature of urolithiasis have resulted in a relatively small decrease in the number of prevalence cases. Furthermore, due to advances in imaging technologies, particularly the widespread use of CT scans and other advanced imaging techniques, the early diagnosis and treatment of urolithiasis have significantly improved (24). Many mild cases of urolithiasis have been promptly identified and treated, preventing the progression of stones and the occurrence of complications. As the level of early diagnosis has increased, the severity of urolithiasis has been effectively controlled, leading to a decrease in ASPR. However, due to the recurrent and chronic nature of urolithiasis, the overall change in the number of cases has been relatively small.
(3) The increase in the prevalence of urolithiasis in women may be influenced by factors such as the ageing of the Chinese population, postmenopausal hormonal changes, lifestyle changes (e.g., diet, obesity), and higher diagnosis rates and health awareness. Women are living longer on average and, as the population ages (21), more women are entering high-risk age groups (e.g., late menopause). Changes in hormone levels in post-menopausal women lead to changes in metabolism and urine composition, increasing the risk of urolithiasis (25, 26). Although the ASPR has decreased as health interventions and treatments have improved in the female population, the prevalence has increased due to the increase in the older female population. In addition, the popularity of diets high in salt, sugar and fat in recent years has led to an increase in the prevalence of metabolic diseases such as obesity, diabetes and hypertension, all of which increase the risk of urolithiasis (27–29).

The Joinpoint regression model found that the age-standardised prevalence rate (ASPR) of urolithiasis for men and women in China will decrease each year from 1992 to 2021, reflecting improved health management, better lifestyles and the development of the healthcare system. As China's economy grows and urbanisation progresses, people's quality of life improves, and the gradual change in lifestyle (e.g., dietary patterns) towards healthier patterns is a positive factor in improving the ASPR, the spread of health education and the improvement of public health policies also provide positive support for the prevention of urolithiasis (22, 30, 31). In addition, advances in the treatment of urolithiasis, particularly the use of minimally invasive techniques (32), have led to more effective disease control, reduced stone recurrence rates and associated complications, and further reduced the overall burden of disease. Furthermore, the model found a significant decrease in ASPR for both male and female urolithiasis, particularly between 2006 and 2009. This positive trend may be closely related to the accelerated reform of China's healthcare system, the expansion of basic medical coverage and the implementation of health policies (30, 31). Improvements in health promotion and dietary patterns, as well as more widespread health education and early detection, helped the population to better prevent and control urolithiasis during this period. At the same time, improved allocation of health care resources as a result of economic growth and urbanisation led to more widespread and timely treatment of urolithiasis, further reducing the burden of urolithiasis. Together, these factors have contributed to the gradual improvement of urolithiasis in China.

As regards the results of the APC model analyses, they can be discussed in three directions: age, period and cohort effects. First, the age effect suggests that the peak risk for both males and females occurs in the 55–59 age group, which is consistent with the previous description of the burden of disease in 2021 and proves the reliability of the results. Secondly, the period effect results are consistent with the Joinpoint regression model, i.e., all ASPRs for urolithiasis are progressively better over time. Finally, the cohort effect showed that the closer the cohort was to 2021, the lower the risk of urolithiasis.

Unexpectedly, despite the gradual improvement in the burden of urolithiasis in China over the past 30 years, the BAPC prediction model suggests that the burden of urolithiasis is likely to increase again in the next 15 years. This apparent contradiction can be explained by several factors. Firstly, the ageing population is a key driver (33). As the elderly population increases, particularly those with declining renal function and increased metabolic problems, the incidence of urolithiasis is likely to rise. Older individuals are more vulnerable to urolithiasis, which contributes to the potential increase in the burden of disease in the future. Secondly, while lifestyle improvements have been observed in recent years, unhealthy dietary habits and lifestyle choices persist in certain populations, particularly in low-income groups and rural areas. These persistent habits may have a delayed effect on the future burden of urolithiasis, potentially counteracting the benefits of recent improvements in public health. Thirdly, the widespread adoption of early screening and diagnostic techniques for urolithiasis has led to better detection rates (24). While this has resulted in the identification of more cases, many of these detected cases may be asymptomatic or only mildly symptomatic, which could explain the apparent increase in the number of diagnosed individuals without a corresponding increase in severe cases. Furthermore, environmental factors, particularly climate change, rising temperatures, and declining water quality, may exacerbate the risk of urolithiasis (34, 35). Water shortages and high temperatures can cause urine to become more concentrated, thereby increasing the likelihood of stone formation, further contributing to the future burden of the disease. Finally, the limitations of the BAPC prediction model itself must be considered. The model assumes that current trends will continue into the future, without accounting for the potential impact of future public health interventions, lifestyle changes, or technological breakthroughs.

Therefore, despite the prediction that the burden of urolithiasis may increase, implementing certain intervention measures remains crucial. For example, enhancing early screening and diagnosis for the elderly population, utilizing advanced imaging technologies (such as CT scans) to improve disease detection rates; promoting health education and improving lifestyle habits to reduce poor dietary practices, particularly in low-income groups and regions with significant urban-rural disparities; optimizing public health infrastructure to ensure equitable healthcare resource distribution between urban and rural areas; improving water quality and raising awareness about water intake, especially in high-temperature regions; and finally, developing targeted policy interventions and health management measures for high-risk groups to mitigate the rising burden.

Although this study uses the latest GBD 2021 database and selects a variety of models for data analysis, there are still some limitations. First, this study analyses data at the national level in China and lacks data from different provinces and regions as well as between urban and rural areas. Due to the large differences between provinces and between urban and rural areas in China, this study would have provided more valuable information for the prevention and treatment of urolithiasis in China if it had been further analysed. Second, the nature of the data in the GBD 2021 database is that they are estimates rather than actual observations. Therefore, the estimates derived from these data modelling methods may be biased.

## 5 Conclusion

In this study, we analysed the trend in the disease burden of urolithiasis in China from 1992 to 2021 by GBD 2021. The results showed that the burden of urolithiasis was significantly higher in males than in females. Furthermore, although the disease burden of urolithiasis has gradually improved for both males and females over the past 30 years, the BAPC prediction model suggests that the burden of urolithiasis is likely to increase for both in the next 15 years. Therefore, prevention, early screening and treatment of urolithiasis in high-risk groups need to be strengthened to respond effectively to a possible future increase in burden.

## Data Availability

The original contributions presented in the study are included in the article/Supplementary Material, further inquiries can be directed to the corresponding author.
